# The Eight-Hour Day in Private Nursing

**Published:** 1919-10-04

**Authors:** 


*To the Editor of* The Hospital.

### The Eight-hour Day in Private Nursing.

*To the Editor of* The Hospital.

Sir,—The problem of the eight-hour working day for the private nurse has within it greater difficulties than appears in the mere description of obvious evils arising from the present length of the hours endured; and this is shown in the absence as yet of any practical scheme or remedy put forward either by the nurses concerned or those who by experience are competent to deal with the question and its manysidedness. The nurse working in a good private practice, under a first-class co-operation, has the remedy largely in her own hands, should depreciation of her service, overwork, or injustice arise. Accountable only in rendering skilled conscientious service and in unquestionable behaviour, she is a law unto herself for her physical wellbeing, and her own welfare, in judging the limits of her capabilities, in the hours of duty, and the concentration of service within them. When the "nerve signs" become apparent, and she has the look of "strained endurance in her eyes," she knows better than anybody else that withdrawal from duty is imperative, and that it is only common-sense to take, at a minimum, twenty-four hours' rest, or a longer interval

**The Editor's Letter-Box**—(*continued*).

before taking a new case. To be in attendance upon a senile mental case for "months at a time," without proper and reasonable intervals of relaxation, is being neither just to the patient nor herself, and in such a case a substitute should be applied for before the physical powers are exhausted. This a nurse is enabled to do (if taking her own fees, or less a percentage), and she can estimate her own powers of endurance, and make provision accordingly, whether her duties or the nature of her case calls for eight or sixteen hours a day. In this respect she must work out her own salvation, as she is under no compulsion if she can show good reason for withdrawal, or to accept a further call immediately upon the termination of a case. Where the call for legislation upon the important question of the eight-hour day for private nurses is most urgent, and where there is exploitation and profiteering out of the toil of the "priceless human instrument," is in the case of those who work for a home or agency at a fixed rate of salary, usually the munificent sum of £40 or £50 per annum. By the fact that their salary is guaranteed them all the year round, so they must work if the calls come, they cannot refuse, even though they may be "filled with a painful lassitude, prematurely aged, and totally lacking in *joie de vivre*." Under such conditions they do their work, expend their efforts, having no choice, while the home or agency to which they are attached takes the guineas from the patients, earned by these unfortunates. For this class of worker, should a scheme of working hours be devised, inquiry and organisation into the conditions of employment of this particular branch of the nursing profession would ultimately result in the righting of very grievous injustices; the actual number of working hours would automatically be placed upon a satisfactory average, although hard-and-fast rules cannot be successfully applied without reacting sooner or later in disadvantages to the nurse or the patient.

Yours faithfully,

"A PRIVATE NURSE."

